# Hierarchical drift diffusion modeling uncovers multisensory benefit in numerosity discrimination tasks

**DOI:** 10.7717/peerj.12273

**Published:** 2021-10-27

**Authors:** Edwin Chau, Carolyn A. Murray, Ladan Shams

**Affiliations:** 1 Department of Mathematics, University of California, Los Angeles, Los Angeles, California, USA; 2 Department of Psychology, University of California, Los Angeles, Los Angeles, California, USA; 3 Department of Psychology, BioEngineering, and Interdepartmental Neuroscience Program, University of California, Los Angeles, Los Angeles, California, USA

**Keywords:** Drift diffusion, Multisensory benefit, Multisensory perception, Cross-modal interaction, Auditory-visual interaction, Hierarchical drift diffusion, Bayesian drift diffusion, Drift diffusion model, Flash illusion, Numerosity judgement

## Abstract

Studies of accuracy and reaction time in decision making often observe a speed-accuracy tradeoff, where either accuracy or reaction time is sacrificed for the other. While this effect may mask certain multisensory benefits in performance when accuracy and reaction time are separately measured, drift diffusion models (DDMs) are able to consider both simultaneously. However, drift diffusion models are often limited by large sample size requirements for reliable parameter estimation. One solution to this restriction is the use of hierarchical Bayesian estimation for DDM parameters. Here, we utilize hierarchical drift diffusion models (HDDMs) to reveal a multisensory advantage in auditory-visual numerosity discrimination tasks. By fitting this model with a modestly sized dataset, we also demonstrate that large sample sizes are not necessary for reliable parameter estimation.

## Introduction

Studies of multisensory integration in perception have by and large been focused on either accuracy or reaction time with regards to decision making (*e.g.*, Leone & McCourt, 2015; Laurienti et al., 2006; Stevenson et al., 2014). These studies usually compare accuracy or reaction time when presented with a unisensory stimulus condition with that of a bisensory stimulus condition, using the difference in accuracy or reaction time as a measure of multisensory facilitation or integration.

However, it is well established that there is a tradeoff between accuracy and speed (Wickelgren, 1977; Heitz, 2014; Zhang & Rowe, 2014). The perceptual system may prioritize one over the other depending on the demands or difficulty of the task. It has also been shown that this accuracy-speed tradeoff may mask the performance improvement when accuracy and reaction time are considered separately (Liesefeld & Janczyk, 2018; Lerche & Voss, 2018). Differences in baseline metrics also often make it difficult to recognize and quantify benefits of audiovisual interactions compared to unisensory conditions, without simultaneously considering task accuracy and reaction time. As a result, it may be favorable to consider both accuracy and reaction time in tandem.

One popular group of models for decision making are drift diffusion models, under which the decision-making process is treated as an accumulation of noisy information (for examples, see Brunton, Botvinick & Brody, 2013; Ratcliff & Smith, 2004). This accumulation of information, or evidence, can be represented as a random walk from a starting point towards one of two choices, conceptualized as boundaries that, when crossed, represent a decision being made (Ratcliff & Rouder, 1998). Due to their simultaneous consideration of both accuracy and reaction time, many have found these models to be better able to uncover multisensory effects compared to more traditional methods of analysis. Specifically, drift diffusion modeling can control for the speed-accuracy tradeoff through its parameters; increasing the amount of information needed to reach a decision can cause the accuracy of responses to increase and the speed of responses to decrease.

**Figure 1:**
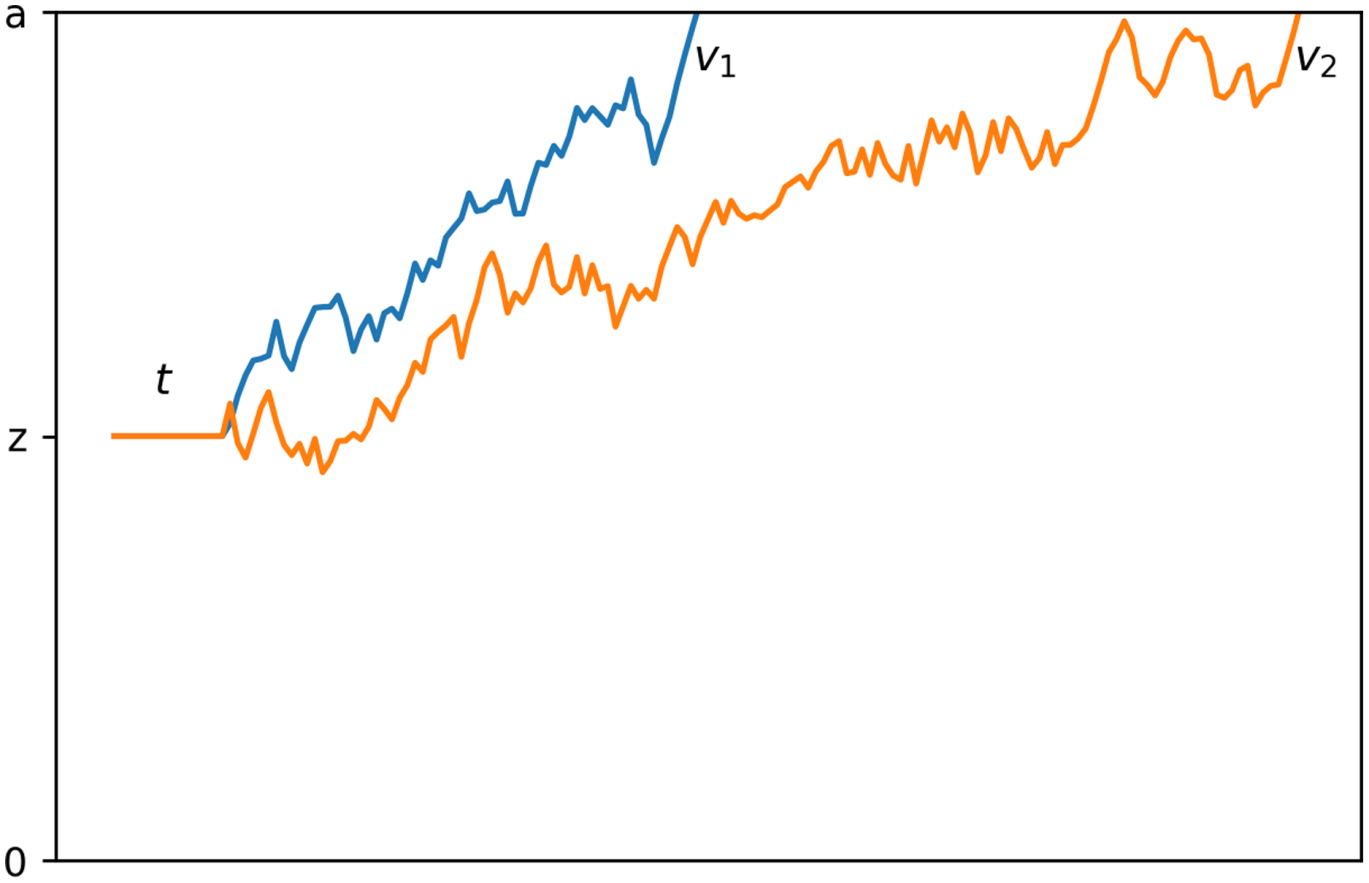
Graphical representation of the drift diffusion process. The process with a faster rate of information accumulation, or drift rate, is depicted in blue.

Drift diffusion models use four main parameters to characterize the decision-making process, which are graphically represented in Fig. 1. First, boundary separation (*a*) describes the amount of information needed to reach a decision; smaller boundary separations indicate faster but more impulsive decisions and larger boundary separations indicate slower, more conservative decisions. Second, drift rate (*v*) describes the rate of evidence accumulation; Low-noise or high signal-to-noise ratio stimuli tend to have a higher drift rate than noisier stimuli. Third, non-decision time (*t*) describes time spent on non-decision facets of a response, including factors such as time required for perception of stimuli and physical movement involved in producing the response. Fourth, bias (*z*) describes the starting point of this process; a bias halfway between the two boundaries indicates the expectation that either choice is equally likely and a bias closer to one boundary indicates that the participant(s) expect that respective choice to be more likely than the other.

Equipped with these parameters, drift diffusion models are able to provide a more reliable and complete characterization of observers’ perceptual processing. However, this family of models are typically too complex and not solvable analytically, therefore requring probabilistic methods to estimate the parameters. Such models thus require large sample sizes to probabilistically estimate robust and convergent model parameters. As a result, the sample sizes of individual participants in many perceptual studies have not been large enough to allow for the application of drift diffusion modeling, which may require hundreds to thousands of trials per participant to converge to a stable solution (for examples, see Milica et al., 2010; Yankouskaya et al., 2018).

However, some groups have recently proposed variants of drift diffusion models that reduce this large sample size requirement to a more manageable number through methods such as iterative simplex minimization (Gómez, Ratcliff & Perea, 2007; Leite & Ratcliff, 2010; Van Zandt, Colonius & Proctor, 2000) or maximum likelihood estimation of fitted parameters (Drugowitsch et al., 2014). Hierarchical drift diffusion models (HDDMs) are one such example, and have recently shown to be able to detect multisensory integration in detection and discrimination tasks where accuracy, reaction time, and sensitivity index (*d′*) failed to detect integration (Murray et al., 2020). This class of models utilizes prior distributions for the DDM parameters which provide a full posterior of each resulting parameter estimate while reducing the sample size needed for a convergent, stable solution.

In the present study, we apply HDDMs to a different type of perceptual task with less data to further investigate the efficacy of this method for detecting and characterizing auditory-visual interactions, and to examine whether they are able to do so more effectively than traditional measures under more common sample size restrictions. We adapted a numerosity discrimination task in which multisensory integration is expected to occur in the multisensory condition. Then by comparing the unisensory condition with the congruent bisensory condition, we examined whether accuracy alone, reaction time alone, and accuracy and reaction time in parallel were able to detect crossmodal interactions. We contrasted the classic accuracy and reaction time measures with those of the HDDMs and compared the insight they provided on multisensory interactions.

## Methods

The experiment was adapted from the temporal task in Odegaard & Shams (2016), where observers were asked to report the number of either flashes or beeps on each trial; a task we refer to as numerosity judgement/discrimination task. Observers were presented with either unisensory visual, unisensory auditory, or congruent auditory-visual stimuli. The performances in the unisensory conditions were then compared with performances in corresponding congruent bisensory conditions to examine the benefit from bisensory processing. For example, the visual performance (judging the number of flashes) in the two flashes and 0 beeps condition was compared with visual performance in the two flashes and two beeps (presented synchronously) condition. The accompaniment of congruent sounds is expected to improve the processing of flashes and the accompaniment of congruent flashes is expected to improve the processing of beeps.

### Participants

Fifteen UCLA undergraduates participated in the experiment, as approved and directed by the University of California, Los Angeles Institutional Review Board in accordance with the Declaration of Helsinki (IRB#13-000476-CR-00003). The participants included 11 females and four males whose ages ranged from 18 to 22 years old. All reported normal or corrected-to-normal vision and hearing. Participants gave their written informed consent to be included in the study and were compensated with course credit. Preliminary analysis of the reaction times revealed that one participant was a strong outlier (exhibiting many reaction times below 10 ms which suggest a lack of effort), thus their data was not included in the analysis.

### Stimuli

Stimuli were presented using a Mac Mini computer, running OS 10.13, *via* MATLAB (MathWorks, Inc., 2014) equipped with Psychophysics Toolbox (Brainard, 1997). Visual stimuli consisted of either two or three flashes of a white disk on a black background on a computer screen. The disk’s diameter subtended 1.5°, and was presented at 2.75° below the fixation point. Fixation point was a white plus sign at the center of the screen. The duration of each flash was 11 ms (one frame), and the SOA was 60 ms (5−6 frames). The visual stimuli were presented on a Sony Trinitron CRT monitor with a 85 Hz frame rate. The auditory stimuli consisted of 2−3 pure tones of 3.5 kHz frequency at a 68-dB sound-pressure level. The duration of each individual beep was 10 ms, and the SOA of beeps was 60 ms. The beeps were presented from two Roland DM-10 speakers symmetrically positioned adjacent to the two sides of the monitor. On trials where both flashes and beeps were presented, the stimuli were synchronized. Synchronicity was verified using an oscilloscope.

### Design

**Figure 2:**
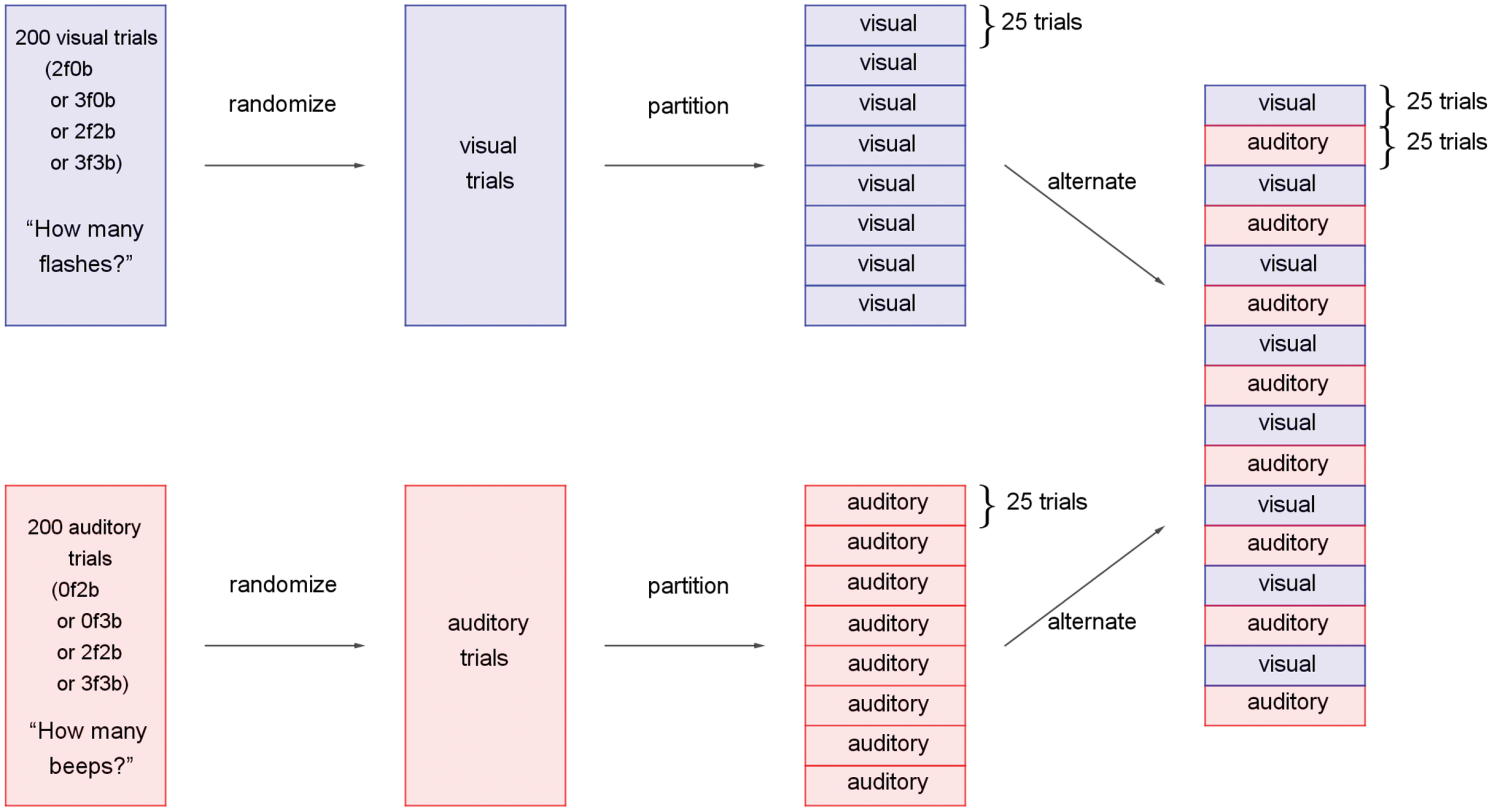
Experimental design for the study. Trials were first separately generated, pseudo-randomized, partitioned into blocks of 25 trials, and interleaved such that visual and auditory blocks alternated throughout the participant’s session.

A within-subjects design was used where all unisensory visual, unisensory auditory, and bisensory audio-visual conditions were presented across trials to each subject. The experiment consisted of 16 blocks of 25 trials each, for a total of 400 trials (Fig. 2). Half of the blocks were “visual blocks”, in which the task of the observer was to report the number of flashes in a two-alternative forced-choice paradigm (“two” or “three”). The other half were “auditory blocks”, in which the task was to report the number of beeps in a two-alternative forced-choice paradigm (“two” or “three”). The visual and auditory blocks alternated, and whether the experiment started with a visual or auditory block was counterbalanced across participants.

During visual blocks, flashes were accompanied by either no beeps, which we will refer to as the unisensory visual conditions, or a congruent number of beeps, which we will refer to as the bisensory visual conditions. The visual blocks, therefore, consisted of four trial types: two flashes and 0 beeps (2f0b), three flashes and 0 beeps (3f0b), two flashes and two beeps (2f2b), and three flashes and three beeps (3f3b). Similarly, during the auditory blocks, the beeps were accompanied by either no flashes (the unisensory auditory conditions: 0 flashes and two beeps, 0 flashes and three beeps), or the congruent number of flashes (the bisensory auditory conditions: two flashes and two beeps, three flashes and three beeps).

### Procedure

Prior to completing the main task, participants completed a practice visual block and practice auditory block, with additional verbal instruction given as necessary. Participants were seated and used a chin- and forehead-rest placed at a viewing distance of 60 cm from the computer screen and speakers that presented the stimuli. A fixation point on the center of the screen was present throughout the experiment. At the start of each block, participants were given on-screen instructions to respond with the number of flashes seen during visual blocks or the number of beeps heard during auditory blocks. After each individual stimulus, participants were also presented with the instruction “How many flashes?” in visual blocks, or “How many beeps?” in auditory blocks, until a response was given *via* key press on a standard keyboard. Key presses for “two” corresponded to seeing or hearing two flashes or beeps, respectively, and key presses for “three” corresponded to seeing or hearing three flashes or beeps, respectively.

### Signal detection theory analysis

Signal detection theory (Wickens, 2001) was used to determine whether the presence of visual stimuli during auditory blocks or auditory stimuli during visual blocks affected the response bias (*β*) and perceptual sensitivity (*d′*) of participants. These two measures were computed similarly to Rosenthal, Shimojo & Shams (2009): *d′* = *z*(*H*) − *z*(*F*) and *β* = 0.5* (*z*(*H*) − *z*(*F*)), where *z*(*p*) denotes the inverse cumulative distribution function of the standard normal distribution corresponding to the response rate *p*. The rates of “hit” and “false alarm” responses are denoted by *H* and *F*, respectively. Cases with *p* = 0 and *p* = 1 were approximated by 1/*N*, and 1 − (1/*N*), respectively, where *N* is the total number of visual or auditory trials. Two flashes/beeps was considered target and therefore, correct identification of two was considered a hit, and a response of two in 3f or 3b conditions was considered a false alarm.

### Hierarchical drift diffusion models

Drift diffusion modeling was performed using the Hierarchical Drift Diffusion Model(HDDM) Python toolbox (Wiecki, Sofer & Frank, 2013). These models use hierarchical Bayesian estimation to solve for both group and individual subject model parameters under the assumption that individual parameters are sampled from the group distributions, creating a posterior distribution and estimate for each parameter at a group level. We will focus on the group parameters in our analyses, and their prior distributions are as follows:

$$a({\rm boundary \ separation}){\rm \sim }{\rm Gamma}(1.5,0.75)$$

$$v({\rm drift \ rate}){\rm \sim }{\rm Normal}(2,3)$$

$$t({\rm nondecision \ time}){\rm \sim }{\rm Gamma}(0.4,0.2)$$

$$z({\rm{bias}}) \sim {\rm{Half - normal}}(0.5)$$

(for more details, see Wiecki, Sofer & Frank (2013)).

Trials with reaction times below 50 ms and above 10 s were excluded and the remaining data was split by block type (visual and auditory). The data from the visual blocks and the data from the auditory blocks were then used to fit two different accuracy-coded models corresponding to the differing tasks in each block. We expect that the visual data and auditory data will have different parameter estimates, and fitting a separate model for each will allow for comparisons between these two blocks. Each model had a boundary separation parameter (*a*), drift rate parameter (*v*), non decision time parameter (*t*), and an outlier term to account for extreme reaction times (*p*_*outlier*_). The bias parameter (*z*) took the value of the prior at 0.5, as the condition varied randomly from one trial to the next. We did not expect boundary separation to differ between unisensory or congruent stimulus trials because trials were randomized; participants were not given any prior knowledge or expectation of stimuli during upcoming trials and therefore should not have differing amounts of accumulated information to reach a decision. As a result, we did not allow the boundary separation parameter to vary with condition. However, we did allow drift rate and non-decision time to vary with condition. This resulted in a total of five parameters in each of the two models (a common boundary separation and varying drift rates and non-decision times).

Two separate models were fitted with the aforementioned model specifications using a single-chain 6,500 sample Markov Chain Monte Carlo (MCMC) simulation with 7,000 samples and 500 burn-in samples. The first model was fitted on the visual block data and the second was fitted on the auditory block data, with each model fitting five parameters. Model convergences were tested with the Gelman–Rubin statistic (*R*_*c*_ < 1.01; Gelman & Rubin, 1992), which compares the between-chain and within-chain variances for each model parameter across multiple Markov chains. We calculated a Gelman–Rubin statistic across five test chains for each parameter to determine convergence, and confirmed that both models converged to stable solutions. We then performed two analyses to evaluate how well the models can reproduce patterns in the data. The first was a posterior predictive check, which checks a model’s ability to recapture the experiment data using the model’s parameters. It does this by simulating a new dataset from input parameters and checking whether the summary statistics of the synthetic data closely match those of the original model. For both the visual and auditory models, 95% credible intervals were able to recapture all summary statistics except the standard deviation of the lower bound. The second evaluation was performing a parameter recovery, which simulates data based on given parameters and fits a new model with the synthetic data. The resulting parameters are then compared to their respective originals, with lower deviances reflecting better performance. Both the visual and auditory models were able to recover parameters with less than 9% deviance from their respective original values.

## Results

**Figure 3:**
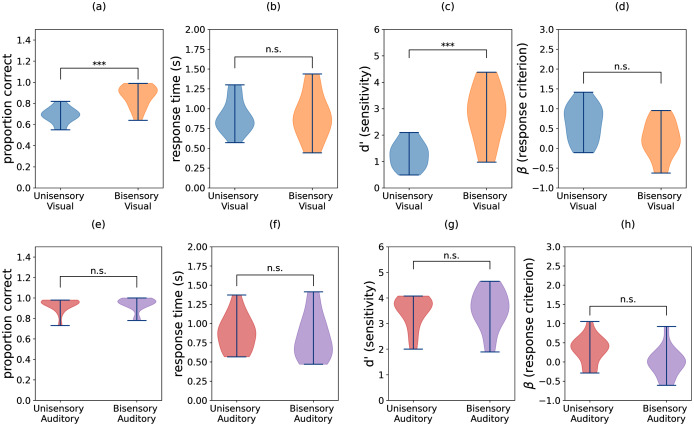
Violin plots showing the distributions of accuracy, response time, sensitivity (*d′*), and criterion (β) for (A–D) visual trials and (E–H) auditory trials. Individual participant means were split between the unisensory visual, (congruent) bisensory visual, unisensory auditory, and (congruent) bisensory auditory conditions. Sensitivity and criterion were computed using trials with two flashes/beeps as targets and trials with three flashes/beeps as distractors. Significant differences in group means are denoted by asterisks (***), and the sample size of each distribution is *N* = 14.

The group means for task accuracy, response time, perceptual sensitivity (*d*′), and response bias (*β*) are shown in Fig. 3. We confirmed that all of these measures have approximately Gaussian distributions. Thus, we performed two-way repeated measures ANOVA with independent variables task (visual *vs*. auditory) and condition (unisensory *vs*. bisensory) for each of the dependent variables. The subscripts “task”, “cond”, and “task-cond” refer to the task, condition, and interaction between the two, respectively. For accuracies, both main effects and the interaction were significant (*F*_*task*_ = 186.712, *p* = 0.000, *F*_*cond*_ = 48.625, *p* = 0.000, *F*_*task*−*cond*_ = 31.140, *p* = 0.000). Similarly for sensitivity, both main effects and interaction were significant (*F*_*task*_ = 89.067, *p* = 0.000, *F*_*cond*_ = 30.749, *p* = 0.000, *F*_*task*−*cond*_ = 19.099, *p* = 0.001). For criterion only the two main effects were significant (*F*_*task*_ = 7.600, *p* = 0.016, *F*_*cond*_ = 8.725, *p* = 0.011). None of the effects were significant for response times (*F*_*stim*_ = 1.572, *p* = 0.232, *F*_*cond*_ = 0.520, *p* = 0.485, *F*_*stim*−*cond*_ = 0.730, *p* = 0.409). We next performed paired t-tests on accuracy, *d′*, and criterion data. The subscripts used are structured as follows: “aud” or “vis” refers to auditory or visual data respectively, “u” or “b” refers to unisensory or bisensory trials respectively, and “acc”, “rt”, “d”, and “beta” refer to accuracy, response time, *d′*, and criterion. The data indicate that bisensory visual trials have significantly higher mean accuracies than their unisensory counterparts (Fig. 3A). This increase in accuracy was deemed significant using a Bonferroni corrected paired t-test for difference in mean accuracy (*M*_*vis*_*u*_*acc*_ = 0.686, *M*_*vis*_*b*_*acc*_ = 0.870, *t*(13) = −6.511, *p* = 0.000, Hedge’s g: −1.957). Similarly, there is a significant difference in *d′* measures between the unisensory and bisensory trials (Fig. 3C, *M*_*vis*_*u*_*d*_ = 1.266, *M*_*vis*_*b*_*d*_ = 2.804, *t*(13) = −5.561, *p* = 0.000, *α* = 0.006, Hedge’s g: −1.890) indicating that the presence of auditory stimuli improved the ability of participants to discriminate between two and three flashes. In contrast, the criterion was not different (Fig. 3D, *M*_*vis*_*u*_*beta*_ = 0.634, *M*_*vis*_*b*_*beta*_ = 0.225, *t*(13) = 2.309, *p* = 0.038, *α* = 0.006, Hedge’s g: 0.832). While there is a benefit in accuracy and sensitivity when comparing multisensory stimuli with unisensory visual stimuli, this was not the case with the auditory stimuli.

As seen in Figs. 3E–3H, the auditory block data does not show a significant difference between unisensory and bisensory trials any of the measures (accuracy: *M*_*audu*_*acc*_ = 0.933, *M*_*aud*_*b*_*acc*_ = 0.939, *t*(13) = −0.696, *p* = 0.499, Hedge’s g: −0.088; *d′*: *M*_*aud*_*u*_*d*_ = 3.455, *M*_*aud*_*b*_*d*_ = 3.529, *t*(13) = −0.505, *p* = 0.622, Hedge’s g: −0.101; *β*: *M*_*aud*_*u*_*beta*_ = 0.316, *M*_*aud*_*b*_*beta*_ = −0.014, *t*(13) = 2.786, *p* = 0.015, *α* = 0.006, Hedge’s g: 0.851). These results indicate that the presence of visual stimuli during auditory trials neither improved participants’ ability to discriminate between two and three beeps nor changed their reaction time or criterion for doing so.

Inspecting individual participant data, we found three distinct categories of participant responses, depending on whether participants benefited in the bisensory condition. Of the 14 participants, five exhibited a slight improvement in auditory performance, which is defined as an increase in accuracy in the bisensory condition without an increase in reaction time. Two subjects experienced a decline in auditory performance, where accuracy decreased without a decrease in reaction time, and seven subjects either experienced an increase in accuracy for the trade-off of slower reaction time or had no change in accuracy. These distinct differences were found to be significant (*F*(4, 1, 6) = 16.278, *p* < 0.001).

Overall, group average data clearly shows a benefit for audiovisual flash discrimination above unisensory visual performance. However, the auditory data is more difficult to interpret. While there are participants who appear to benefit from the addition of visual stimuli, half or more of them do not show the same changes in accuracy and/or reaction time. Furthermore, the addition of visual stimuli do not significantly affect sensitivity nor response bias. By looking only at traditional measures alone, there is no clear benefit of multisensory stimuli over unisensory auditory stimuli.

### Model parameters

**Figure 4:**
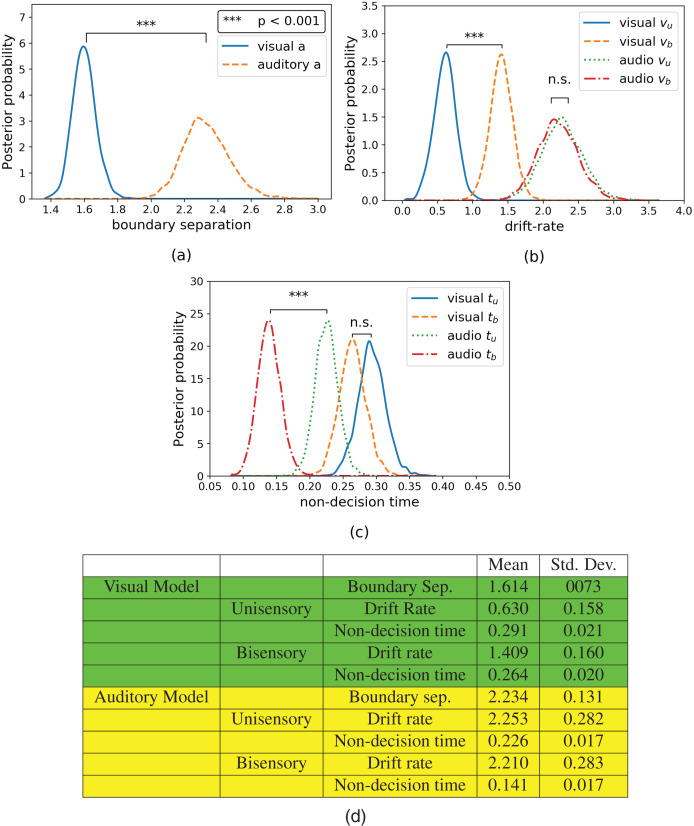
Posterior plots for (A) boundary separations, (B) drift rates, and (C) non-decision times and their respective (D) means and standard deviations. Boundary separations are common between unisensory and bisensory blocks, thus there is only one boundary separation per model. Unisensory parameters are denoted with a subscript “u” and congruent parameters are denoted with a subscript “b”.

Hierarchical drift diffusion modeling was applied to both visual and auditory discrimination task datasets. The resulting parameter posteriors for boundary separation, drift rate, and non-decision time can be seen in Fig. 4. The subscripts have the same meanings as before, with the addition that “a”, “v”, and “t” refer to the drift diffusion parameters: boundary separation, drift rate, and non-response time. Note that boundary separation “a” does not differentiate between “u” or “b” due to the combination of these trials in a single common parameter (see Hierarchical Drift Diffusion Models subsection in Methods section). As shown in Fig. 4A, the boundary separation of the visual data (*M*_*vis*_*a*_ = 1.616) was significantly lower (*t*(13) = −8.2, *p* = 0.000, Hedge’s g: 9.688) compared to that of the auditory data (*M*_*aud*_*a*_ = 2.329).

The drift rate estimates can be seen in Fig. 4B. In the model for visual data, the drift rate of the unisensory trials (*M*_*vis*_*u*_*v*_ = 0.633) is significantly lower (*t*(13) = −4.973, *p* = 0.0002, Hedge’s g: 4.952) than that of the bisensory trials (*M*_*vis*_*b*_*v*_ = 1.412). In the model for auditory data, while the drift rate of unisensory trials (*M*_*aud*_*u*_*v*_ = 2.261) is slightly lower than that of the bisensory trials (*M*_*aud*_*b*_*v*_ = 2.207), this difference was not significant (*t*(13) = 0.575, *p* = 0.575, Hedge’s g: 0.153).

Finally, the non-decision time estimates can be seen in Fig. 4C. The non-decision time for the unisensory visual trials (*M*_*vis*_*u*_*t*_ = 0.291) is greater than that of the bisensory visual trials (*M*_*vis*_*b*_*t*_ = 0.264), although this difference is not significant (*t*(13) = 2.043, *p* = 0.0618, Hedge’s g: 1.356). However, the non-decision time for the unisensory auditory trials (*M*_*aud*_*u*_*t*_ = 0.233) is significantly greater than its bisensory counterpart (*M*_*aud*_*b*_*t*__= 0.154_, *t*(13) = 10, *p* = 0.000, Hedge’s g: 4.955).

## Discussion

Accuracy and sensitivity (as measured by Signal Detection Theory index of *d′*) did show a benefit of multisensory stimuli in visual performance. However, none of the traditional measures indicated multisensory benefits in the auditory performance. On the other hand, HDDM was able to reveal a benefit of multisensory stimuli in both visual and auditory performances. This finding is consistent with our previous findings showing that drift diffusion modeling provides a more sensitive measure of multisensory integration benefits (Murray et al., 2020).

Importantly, HDDM also characterizes the aspect of processing that benefits from integration in each case. Namely, here the visual processing benefits from congruent beeps in accumulation of evidence can be considered as improved sensitivity (Ratcliff, 2014). This is consistent with the findings of Frassinetti, Bolognini & LÃ davas (2002) that perceptual sensitivity to visual stimuli increases when two visual and auditory stimuli are overlapping and spatially consistent. While the auditory processing does not show a benefit in drift rate (sensitivity), this could be due to the fact that the drift rate is already very high; perhaps reflecting a ceiling effect. This lack of benefit may also be due to the lower sensitivity of drift diffusion models with the relatively high accuracy of auditory trials. However, the auditory task also required more evidence to reach a decision, as shown by a greater boundary separation in Fig. 4A. This suggests that participants were more cautious or conservative with decision-making during the auditory task. On the other hand, the higher drift rate also suggests that the task was less difficult than its visual counterpart. Finally, the non-decision time for auditory processing appears to benefit from the accompaniment of congruent flashes. While it has been found that visual stimuli can improve the response time of auditory speech perception (Paris, Kim & Davis, 2011), the evidence for similar effects in more general perceptual tasks is more sparse. This finding suggests that visual stimulation contributes to a more efficient auditory response.

These findings are more informative about the effect of auditory-visual interactions on perceptual decision-making than what could be gathered by raw accuracy and reaction time data alone. Furthermore, the HDDMs were able to account for the data despite a fairly small sample size. Therefore, these results are encouraging for the usability of HDDMs, in that studies typically gathering small to moderate sample sizes could likely utilize HDDMs to create a more complete picture of perceptual processing across a variety of tasks.

## Supplemental Information

10.7717/peerj.12273/supp-1 Supplemental Information 1 Participant responses to individual experimental trials. Each file contains the experiment parameters for an individual participant. The respMatF and respMatB variables contain the responses for visual and auditory trials, respectively, where “90” represents a response of “two” and “91” represents a response of “three”. The resptimeF and resptimeB variables contain the reaction time in seconds for visual and auditory trials, respectively. Finally, the trMatF and trMatB variables contain the stimuli for visual and auditory trials, respectively, where the first column represents the number of flashes and the second column represents the number of beeps.

## Additional Information and Declarations

### Data Availability

The following information was supplied regarding data availability: Data for visual responses is available at Github: https://github.com/chauedwin/NumerosityHDDM/blob/master/vis_response_data.csv Data for auditory responses is available at Github: https://github.com/chauedwin/NumerosityHDDM/blob/master/aud_response_data.csv Code for data analysis and HDDM modeling is available at Github: https://github.com/chauedwin/NumerosityHDDM/blob/master/ExpResults.ipynb.
